# Selections and Abstracts

**Published:** 1916-09

**Authors:** 


### SELECTIONS AND ABSTRACTS

### MEDICAL CARE OF THE NATIVE ALASKAN

The problem of caring for the natices of Alaska is among the most difficult matters which confront the government in its relations with the aboriginal tribes.

There is no central point in Alaska, Seattle being the trading centre of the Territory.

These people are scattered along a waterfront of more than 5000 miles. They live in small villages. They are still influenced by the superstitions which have come down to them from the centuries. They hide, rather than seek relief for their ailments, believing that there is some divine retribution in misfortune.

Secretary Lane of the Interior Department, who personally knows every part of Alaska, has given tender consideration to the needs of the native Alaskan, and great improvement has taken place in the care of these people, especially during the past two years.

Syphilis and tuberculosis, here as elsewhere, have wrought sad havoc with the primitive people.

The editor of the Medical Sentinel, in a trip just completed in Alaska, was forcibly impressed by the special interest now being shown by the government in the medical side of care for the natives.

At Juneau, Dr. Douglas Brown, a recent arrival is in charge of a splendid native hospital just completed by the Interior Department, which looks after fourteen near-by villages. Dr. Brown serves under the Educational Division of the Interior Department, is a civil service employe and was for some years with Col. Gorgas on the Panama Zone.

At Haines a special hospital is soon to be erected for tubercular cases and soon a colony with every modern equipment will be in operation.

In other portions of Alaska, seven or eight physicians have been put in charge of the medical Indian service, and three other small native hospitals are already maintained by the government in the territory.

An attempt is now being made by Secretary Lane to employ teachers in the Educational Division, for stations where no doctors are located, who are also trained nurses. These teachers have some special training for emergency medical work, are given a medical and surgical equipment of simple character, and provided with proper instructions for the service along medical lines. As fast as appropriations can be secured, district zone are being organized comprising a neighborhood of native villages, for which a general hospital and a competent physician is supplied.

The insane native has the benefit of care outside of Alaska, where, in a milder climate, the percentage of recoveries is very large. The tubercular insane live in a separate department, at Portland, Oregon, where they enjoy every qualification for modern treatment.

The Educational Department in these more recent departures, seeks, among other things, to educate the natives as to the prevention of tubercular infection. Also as to the dangers of syphilis, its possible cure under appropriate treatment, thereby effecting the lowest possible evil to the living, as well as to the unborn progeny of the natice races of Alaska.—Medical Sentinel.

In short pithy sentences, an American Ambulance driver, a Harvard student, has contributed to the October number of the American Red Cross Magazine a thrilling account of ten days' experiences before Vardun, immediately preceding the Somme engagements. He tells a story of the rescue of wounded soldiers on the French side, in the midst of terrific sharpnel and gas fighting, so graphically that an editorial note says it "exceeds in vividness and action anything bearing on individual achievements that has come to our hands from the Western war front in Europe".

This heroic young American, a member of the Harvard section which was later honored with military medals and crosses of war, "rolled" for ten nights in the light auto ambulance bringing to field hospitals badly wounded "blesses." He describes plunging into gas clouds, wearing a gas mask, with sharpnel shells whistling and exploding about his ear and of rescuing at one time one of his fellow ambulance men, the whole back of whose car had been shot away. This man he found in a pitiful plight, his nerves gone and unable to talk straight. With his car full of wounded men and shells exploding all about one of the rear wheels of his ambulance became entangled in barbed wire. His frantic efforts to unwind this wire, now and then ducking under his car because of the warning whistle of a shell, are so realistically described that the reader might easily imagine being at his side. The explosion of one shell threw him on his face in a pool of horse's gore, and another shell blew out both of the rear tires of his car so that one trip back was made on the rims. The roadway, he says, was literally paved with dead horses and men and wreckage.

"The far-sung 'Charge of the Light Brigade' has been many times outdone by men afoot, men in automobiles, and men in the air since the great war began," say the Magazine. "And is it not inspiring especially to read of the employment of such matchless bravery to save the lives of fellow-men, as numerous boys, scarcely out of their teens, are doing in Europe today?

Now for a dash into the black, reeling, maddening, brobdingnagian battle of the ages with this Harvard lad in a shell-battered ambulance and to learn of his reward. You'll be proud of him and of all like him."

Among other articles and feature stories, most of which are illustrated with photographs, the October number of the Red Cross Magazine carries the second instalment of the Civil War reminiscences of Mr. James Tanner, of Washington, Register of Wills of the District of Columbia, who was a corporal in the 87th New York Volunteers. He lost both of his feet in the second battle of Bull Run and suffered unspeakable miseries therefore due to the woeful inadequacy of the medical and nursing system. Corporal Tanner's story abounds in patriotic, pathetic and humorous incidents after his great misfortune and points the way vividly to the need of a potential Red Cross organization. Veterans of the Civil War as well as later generations will find much of interest in his portrayal of life in a Civil War hospital.

Due to the rapidly increasing cost of paper and the constant desire of the Red Cross to operate economically, the October number of the Red Cross Magazine is somewhat reduced in size. The circulation of this issue is announced to be 230,000.

---

### 125 RED CROSS NURSES SENT TO MEXICAN BORDER.

To reinforce the Army Nurse Corps on the Mexican border the War Department has obtained from the American Red Cross 125 graduate Red Cross enrolled nurses for assignment to camp and base hospitals distributed along the frontier from Nogales, Ariz., to Brownsville, Tex. Fifteen of these 125 are representatives of big civil hospitals in which American Red Cross base hospital units have been organized by Col. Jefferson R. Kean, Medical Corps, U. S. A., the Director General of Military Relief of the Red Cross. The nurses were selected by Miss Jane A. Delano, Chairman of the National Committee on

Red Cross Nursing Service, whose department has over 7,000 enrolled graduate nurses.

While Colonel Kean has organized twenty-five base hospitals, eight only of these will be represented in this detail of 125 nurses. The names of these fifteen nurses from the eight hospitals, the name and address of the hospital, and the point or camp in the Southwest where the nurses are assigned, follows:

**Representatives from Red Cross Base Hospital Units.**

Harper Hospital, Detroit, Mich., to San Antonio, Tex.—Mary DuPaul and Donna Sutliff.

Rochester General Hospital, Rochester, N. Y., to Nogales, Ariz.—Ruth M. Randall and Hazel Vegiard.

Post Graduate Hospital, New York City, to El Paso, Tex.—Isabelle MacMaster and Gertrude Williard.

New York Hospital, New York City, to San Antonio, Tex.—Edith A. J. Howard and Marie K. Falconer.

German Hospital, New York City, to Deming, N. M.—Katherine P. Duelle and Clara L. Horn.

Boston City Hospital, Boston, Mass., to San Antonio, Tex.—Belinda Scanlan and Della M. Currier.

Massachusetts General Hospital to Laredo, Tex.—Leonor A. Field.

Cincinnati General Hospital to McAllen, Tex.—Cynthia Richardson and Elsie Magnus.

**Nurses Selected from Other Points.**

Graduate enrolled Red Cross nurses selected from other points and the places assigned, follow:

Denver, Colo., to Nogales, Ariz.—Margaret B. Otis, Virginia Carnahan, Eva L. Fortman, Margaret F. Hamilton, Harriet H. Hart, Mae E. Walton, Mabel Light, Elizabeth M. Long, Helen M. Dixon and Lenora Rall.

Philadelphia, Pa., to Brownsville, Tex.—Edith L. Wood.

Newark, N. J., to El Paso, Tex.—Linda K. Meirs.

Cleveland, O., to Deming, N. M.—Ina May Clark and Mrs. Marie A. Shields.

Canton, O., to Deming, N. M.—Mabel Firestone.

Birmingham, Ala., to McAllon, Tex.—Eida E. Peterson, Mae Rowan and Mattie Hinson.

Augusta, Ga., to McAllen, Tex.—Emma L. Dozier and C. Elizabeth Thomas.

Buyalo, N. Y., to Brownsville, Tex.—Dora Scheuer, Vera V. Dunkle, Mildred Engeland, Grace C. Hammond and Jessie B. Mallory.

Selma, Ala., to McAllen, Tex.—C. Marie Hansen.

Dayton, O., to Demong, N. M.—Cora V. Moore.

Commerce, Ga., to Laredo, Tex.—Katheryn Crowley.

New Orleans, La., to Eagle Pass, Tex.—Lottie Glazener, Mary Ann Kief, Mary P. Little and Maude F. Mims.

Denver, Colo., to Eagle Pass, Tex.—Anna May Barr.

Camden, N. J., to El Paso, Tex.—Laura E. Wilde, Emily Jummel.

Sioux City, Ia., to Llano Grande, Tex.—Nellie M. Porter, Katherine Aten, Edith G. Becker, Florence Griswold, Catherine Hoffman, Laura G. Leeder, Marie S. Ohge, Jenny C. Robertson, Cecilia Strief and Abbie Taber.

Crookston, Minn., to Llano Grande, Tex.—Hilda Twedten and Ida E. Twedten.

Baltimore, Md., to Eagle Pass, Tex.—Florence P. Kennedy.

---

### GLENARD'S DISEASE.

By Bradford C. Loveland, A.M., M.D., Syracuse, N. Y.

(N. Y. State Journal of Medicine.)

Glenard's disease, abdominal, or visceral ptosis, is no new disorder, and yet the last word has not been spoken upon it, and may not be for a long time. In fact, like the poor, it is always with us, and this is not intended as a pun, even if many of these patients are quite emaciated.

The symptom complex was described a number of years ago, and while I shall consider it as a condition rather than a disease, I do not wish to overlook the complex character of its symptoms, or its fundamental causes, and I am persuaded that only a feature or two are recognized by the physician, and that feature is dictated more by the point of view from which the physician sees the patient than the importance of the feature in question.

It is my purpose to review from the standpoint of experience and observation this condition, and to briefly outline what has been in my hands a successful method of treatment in many cases which had survived often long periods of treatment of various sorts, with at best indifferent results, before they came under the regime referred to.

First then the condition: A poorly nourished patient (not necessarily anaemic), usually under weight, muddy complexion, with rings under the eyes, complains of tiring easily, headache, gas in stomach after eating or even while eating. Appetite poor, or if hungry is satisfied before a reasonable amount of food has been eaten, complains of cold extremities, especially at night, usually constipated bowels and on inquiry we will often find insufficient action of kidneys, sleep broken and dreamy and to these symptoms are often added palpitation or tachycardia, depression, a mental state of apprehension as of some impending evil and not infrequently in the case of mothers, a feeling as if they could not stand the prattle of the children.

Diagnosis.—It should not be necessary to say that a careful examination of some sort is absolutely necessary if this condition is to be recognized, even though the symptoms and signs observed in a casual way may point in this direction, but I am sure that many of these cases are all about us complaining now and then to a physician when their burden becomes unbearable, but impressing the physician only sufficiently to prompt the suggestion of a vacation for rest, and perhaps a tonic or laxative, nothing further; and little wonder that such a patient becomes discouraged in the effort to get relief and for a time gives up treatment, only to be forced to another trial when she can no longer bear the constant distress.

There are more ways than one to get the information we need about the patient's physical condition. The X-Ray bismuth test can outline the stomach or the colon very clearly, but is open to some possible errors even in these functional cases, first due to the increased peristalsis incident to the laxatives given frequently to prepare the intestinal tract for the bismuth paste and, second, due to the mechanical result of the weight of the bismuth paste ingested, but withal may in a good measure be depended upon, although an X-Ray picture taken as it usually is with the patient in an erect posture, conveys a different idea as to the shape of the stomach and the sharp angels of the colon, than one gets from the percussion methods with the patient lying on the back.

It may be borne in mind that all our anatomical plates have been made from cadavers lying flat and I have no doubt that they do not represent the relations of the organs as they would appear in a normal body in the erect position.

The late Profesor Ford, of the University of Michigan, once told me that in dissecting over 2,000 subjects, he had never found a misplaced kidney, yet we are all aware that a displaced kidney is of frequent occurrence during life.

The tube is rarely necessary for diagnosis in these cases and should never be used for any other purpose except diagnosis and the acute necessity of relieving the stomach of a poisonous content.

The method most simple, most generally available and sufficiently accurate for almost all purposes, combines inspection of the abdomen in the uncovered state with the patient in the erect and prone position, which will often show a flat abdomen in the prone position and occasionally we will find the walls so thin that the peristaltic movements may be observed and some of the viscera outlined by the ordinary vision, and a pendulous abdomen with a depression at the epigastrium, when the patient is erect.

The next step is palpation, which will help locate tender spots and eliminate to quite an extent the presence of new growths, will give us the location and size of the kidneys if displaced, and often give much information as to the stagnation of foecal matter in the colon. But most valuable, perhaps, is percussion aided by auscultation or auscultatory percussion. This method will give the outline of the stomach, liver, spleen and often the colon, besides the interesting and helpful knowledge of the heart and lung conditions, and quite as constant results are obtained as with the bismuth test, though the stomach does not assume quite so much of the boot shape.

The result of this examination will eliminate organic diseases, recognize the dilated and sagged stomach often below the umbilicus, a movable kidney, a colon carried down by the lower border of the stomach, the soft, flabby, abdominal muscles and above all the neurasthenic foundation, which is most cases is inborn. This complete physical inventory not only furnishes a diagnosis of the condition, but a basis for treatment.

Treatment.—Historically, the treatment has passed through many phases based upon as many theories or conceptions. The aim has been to correct, relieve, or support principally the sagged stomach. A once popular method was to feed the patient a small amount of concentrated food, meat and bread largely, and a small amount of water, hoping the stomach would contract to fit its contents.

Not a few have recommended gastro-enterostomy and making a reef in the stomach, stitching the kidney back or removing a portion of the colon, some or all of these.

More yet have apparently despaired of cure and adopted either a supporting corset, or some form of a belt, being content to support the ptosed organs by an artificial method. None of these have been satisfactory for none have attacked the condition from the direction of its causes.

Etiology.—The fundamental cause of abdominal ptosis, or at least the most frequent cause is heredity. That is, most of these patients were born with a poor nervous endowment, and if we take up the causes in the order of the development or growth of the patient as well as the frequency, we must recognize next rapid eating and over filling the stomach during early childhood.

I believe that abdominal ptosis, or at least gastroptosis, is very frequent in children from the fifth to the twelfth year, usually unrecognized and generally coming back to normal as the child takes up more athletic exercise or an occupation which develops stronger adbominal muscles.

Next in order comes the period of adolescence, when the young person is acquiring the sedentary habit and developing latent neurasthenia and upsetting digestion in the endeavor to keep pace with the high school hours and examinations.

It is during this period when the nervous mettle of these poorly endowed, though often brilliant persons, receives its first severe strain, and not very infrequently it proves unable to meet it. They drop out of school for a term of two, going back feeling well, but simply because the strain has been taken away, but there is no more endurance than before. A little later another term is dropped, or the whole course is given up.

Next comes the period when the man takes up the responsibility of a business or profession, full of ambition, and again finds the weight of work and worry too great for the physical endurance, or in the case of the woman the cares of the home, and especially the strain of child-bearing bring about a similar result, in this case added to by the mechanical distension of the abdomen, which does not return to the normal because the mother thinks she should be up and caring for her baby as soon as other mothers, not realizing her poor physical endowment, and consequently doing little or nothing to improve it.

Now the distresses reach a point which finally drives the patient to the physician, with varying results.

Treatment.—The treatment then which will prove successful must take into consideration the nervous constitution and the inefficient muscular support brought out by the physical inventory, and must aim to increase the physical endowment and the abdominal muscular strength, and I will outline the treatment under the heads if Dietetic, Hygienic, including Electrical, and Medicinal; hence you will see it involves a system rather than single idea.

Dietetic.—As most of these patients are thin and weak as well, a more or less forced diet is necessary, and because the appetite cannot be trusted as a guide, each meal, and lunches if required, should be ordered by the physician, giving specific attention to every detail, amount, variety of food and drink, and the time of each feeding. To make clear what I mean I will give a sample prescription of diet for a typical patient, five feet five inches high, weighing ninety-five pounds, which is about an average type, and who has never weighed more than 105 pounds:

Breakfast: 8 A. M. Soft boiled or poached egg, a slice of bread or a gem. A dish of oatmeal mush or crushed wheat, well cooked, salted, but not sweetened, milk on cereal, and close the meal with one full glass of milk and a half glass of water, often better mixed.

At 10:30 A. M.: Lunch of one glass of milk, a raw egg and a half glass of water. Egg may be taken in the milk, or better still, swallowed just as it comes from the shell, and the milk taken afterward.

Dinner: 1 P. M.: One lamb chop or a similar amount of beef or chicken. Mashed or boiled potatoes, peas or string beans. One slice of bread. Tapioca, custard, plain rice pudding, blanc mange, apple sauce, peaches or pears, ripe olives, and one glass of milk, with a half glass of water to finish up with.

Lunch: 3:30 P. M. Same as at 10:30 A. M.

6 P. M.: Supper much the same as breakfast, except that a dry cereal like shredded wheat or maple flake may be used. 9 P. M.: Milk and egg with water the same as other lunches. One glass of water, hot or cold as desired, half hour before each meal, and one glass of water about an hour after each meal.

This diet may produce a feeling of distension for a short time after each meal at first, due to the increased intra-gastric pressure induced, but this will force the absorption of the gas which up to the meal time has lain passively in the stomach, and will insure a gain of one and one-half to two pounds per week in the patient.

After a few days the feeling of distension after eating will disappear and the constipated habit will also be relieved.

Hygienic and Electrical.—A cool sponge bath over the body or at least over the abdomen on rising, followed by brisk rubbing and calisthenics as follows: Erect posture, flex and extend arms, flex and extend legs. Bend forward and backward. Bend one side, then the other. Squat down and get up. Lie down, and elevate one limb and then the other as far as possible with the knees straight. Deep breathing, beginning with lungs emptied and hands down at sides. Inhale as the arms are reached way beyond the head as far as possible. Exhale as hands are replaced at sides. Three or four exercises each, increasing one each after patient gets so the above is not tiresome.

Walk twice daily in the open air to such extent as patient can stand, say a half mile, and an hour rest after dinner lying down.

The electrical treatment suited to this case is continuous current three or four milliamperes positive electrode at occiput and slowly moved down to or a little below a line drawn between the lower points of the shoulder blades or scapulae. Negative electrode to the base of the spine. Time, three or four minutes. Now with the negative electrode or Faradic current, applying the other electrode over the abdomen from right to left in the direction of the peristaltic waves. Strength sufficient to make positive muscular contractions whenever the electrode passes over the contractile points. Time, six to eight minutes. Every second day.

Medicinal Treatment.—This is not so important as the regime already described, but frequently some mild stimulation of the liver and tonic for the stomach and bowels is helpful, so we will give this average case Hydrarg. protoidide one-eighth grain before each meal for a while, and a tablet of extract nux vomica one-fourth grain, rhubarb and soda of each one grain after each meal.

The results to be expected from these measures are a gain of one to two pounds in weight per week. A gradual improvement in the bowel movements, a feeling of improvement, better rest nights, and a gain in strength, in proportion, and in from two to three weeks a marked change for the better will occur in the ptosis, and a decided increase in the strength of the abdominal muscles.

It is not only safe to make such a prediction, but it is a wise thing to tell the patient what may be expected, as it helps to secure co-operation.

Then, too, when these prophesies begin to be fulfilled, your patient's hope and courage and determination grow apace.

I will only lengthen this paper sufficiently to refer to you or three cases which are fair illustrations of what can be done for these chronic sufferers.

Mrs. W. D. N., thirty-two years old, a chronic invalid for thirteen years, mother of one child ten years old. Came to my office November 30, 1900. She suffered from weakness, headache, constipation, scanty and painful menstruation, tachycardia or palpitation, and worst of all, a feeling of apprehension of sudden death, which so controlled her that she dared not stay alone in the house much less go out alone.

Examination showed her stomach to reach a point a hand width below the navel, right kidney palpable just above the pelvic brim, colon festooned and uterus displaced downward and backward.

She was treated according to the plan already described, with the result that her symptoms disappeared, and on March 2, 1901, my diagram showed her stomach and kidney about in their normal position. On December 15, 1911, she came to see me again for neuritis in her arm, and I requested an examination of her abdominal organs for comparison with the earlier diagram. This showed her organs in normal position as when she left my care in 1901, and she said she had had no symptoms referable to her old trouble since she left my care.

This case was reported more fully in a paper read in 1911 at the New York State Medical Society, entitled, "Aften ten years: a record of experience with abdominal ptosis."

Another case in which the diagnosis of the stomach position was confirmed by observation on the operating table may be reported.

Mrs. C. D. S. came under my care March 30, 1903. I will not go into the details of all her symptoms, which were much like I have described as a typical case, but my examination showed her stomach as low as the brim of the pelvis, and a prolapsed uterus badly lacerated from child birth (she had two children), and on the advice of a surgeon she went into the Women's and Children's Hospital, October 6, 1903, and was operated for repair of the cervix and ventral surpension of the uterus.

At this operation her stomach presented in the wound and it was difficult even in the Trendelenburg position to keep it back enough to have the field free for the suspension operation.

The eporation was a success from the surgical standpoint, as the patient recovered. A rather protracted course of treatment was required to get the patient in good condition.

Another operation for hysterectomy in 1908 showed a practically normal position of the stomach, and with ups and downs due to a very strenuous life, she had been and is pretty well as compared to what she was for years previous to 1903.

Another case briefly reported will be sufficient to illustrate what I have said in this paper.

Case 3.—Mrs. C. C. D. Consulted me November 25, 1908. She was married, mother of one child eleven years of age, of good heredity. She dates her illness to a nervous breakdown ten years ago from which she had never recovered. She complained of gas, palpitation so that she frequently had to sit up at night for a while. Of headaches, constipation, numbness in limbs and arms, weakness, etc.

Examination showed the lower border of the stomach about two inches below the navel, the right kidney displaced downward about four inches, and the other evidences of general ptosis of the abdominal contents, and weight one hundred and three pounds.

The treatment combined the regime described, and on February 1, 1909, my diagram showed great improvement in all the conditions, including the position of the stomach, weight 109 1-2 pounds. The following September 23d she showed weight 120 3-4 pounds and the stomach about in normal position.

I have known the patient and cared for her when she needed medical attention since that time, and to the present have only been asked to prescribe for occasional or transitory ocnditions.

The prescribing of such a regime may seem easy, but its success depends on experience and the courage and conviction to secure the co-operation of the patient, and the physician must see the patient frequently enough to keep up his co-operation and courage until he can see results. Then things will move on more smoothly and less personal attention will be required.

---

### VERIFY YOUR REFERENCES—A WORD TO MEDICAL WRITERS.

By Frank Place, Jr., New York.
Assistant, Library of the New York Academy of Medicine.

Dean Burgon (1), in his life of Doctor Routh, tells the story from which I take my text· Mr. Burgon, then a recent graduate of Oxford, called one day upon the venerage president of Magdalen College, Doctor oRuth. His account proceeds: "I believe it was then that I ventured to address him as follows: 'Mr. President, give me leave to ask you a question I have sometimes asked of aged persons, but never of any so aged or so learned as yourself.' He looked so kindly at me that I thought I might go on. 'Every studious man, in the course of a long and thoughtful life, has had occasion to experience the special value of some axiom or precept. Would you mind giving me the benefit of such a word of advice? He bade me explain—evidently to gain time. I quoted an instance.

He noodded and looked thoughtful. Presently he brightened up and said, 'I think sir, since you care for the advice of an old man, sir, you will find it a very good practice (here he looked me archly in the face) always to verify your references.' "

Verify your references. That is my text, and in the present instance I wish to direct this text particularly to the medical man who would venture into print. It is to be assumed that he has fulfilled Bibling's (2) four rules for the preparation of a medical paper. 1. "Have something to say· 2. Say it. 3. Stop as soon as you have said it. 4.Give the paper a proper title." It is in the revision, the polishing off process, that he should, among other things, go over his list of references and verify them. To direct attention to this phase of authorship is the purpose for which this paper is written.

The science and art of medicine is so dependent upon its literature that reference to authorities is a recognized part of medical composition. The quantity of such printed matter is very great and quotation of sources is as necessary in medical literature as in any other field of literary endeavor. Such is the indifference on the part of writers, however, to the place and importance of the bibliographical reference that some attention should be directed to it.

What underlies that admonition, Verify your references, that makes it the only advice offered to a studious soul seeking help? It is of the spirit of the scientific method. Substantiate your statement by proof, either of your own or by the work that others have done before you. We work with the tools that others have made and placed in our hands, and we hope to make tools to place in the hands of others who follow us. If our predecessors have experimented and have left no record in material objects or written description, their works profit us nothing. For the purpose of storing up medical advance in deed and thought medical books and journals exist, and in them we shall find description of the processes of work that others have employed· To find these papers in the great mass of literature, indexes and catalogues were invented. In addition, most writers, in reporting their work or ideas, read and gather the publications that relate to their particular task; and then, to help others in the same line,, to record their own research, and to have a line of defense against criticism, they print with their report a list of the papers they have consulted. Should not the scientist be as truthful and as accurate in recording his help as in giving his own work? One says "yes" without hesitation. How, then, can the author permit references to be printed which are not only false as to the fact, but which also seem to be intentionally so? Some so far forget science as to quote articles that it is plain that they have never seen, but have lifted bodily from some other list. Certainly there should be some distinction between the article read and the one known only by hearsay. If an author would put himself in the place of one of the audience whom he is addressing; if he would but read the paper and verify the references as if he had never heard of the subject before, then he would begin to realize some of the deficiencies of his paper and to appreciate the need for fullness and accuracy of statement in his bibliography; he would perceive the reasons for including data whose usefulness he had never before recognized.

It is hard to understand the point of view of the writer who is preparing a paper and who says: "No one will ever take the trouble to look up these references. They are near enough correct now. Let them look 'em up as I had to." The obvious reply is, "Why print your references at all then? If you are so little interested in your work as that, if you have so little purpose to help others, why do you even print your paper? There aer plenty of papers waiting to be published that are just as good or better." The time an author spends in verifying his references is more than saved for every one of his readers. "Rong References" are frequently so very wild that it is hardly worth while to continue the search for them (3). The author. who knows the material, can more easily verify and correct his references before publication than any reader, "each in his separate sphere," can spend hours or days in finding them afterward. Nothing in science is too insignificant to notice. Therefore, verify your references.

The experience of writers and bibliographers has shown that the efficient bibliographical reference is the one containing the complete and correct answer to the questions, "Who wrote it? What is it about?, When are where was it published?" Answering these questions the citation should stand as it does in the Index Medicus and in the Index-Catalogue of the Library of the Surgeon-General's Office. In referring to a book the details are these: 1. Author's name with initials. 2. Title of book. 3. Edition, other than the first. 4. Place, publisher and date (the imprint). 5. Volume, and page therein if a particular statement is to be quoted. On the other hand, a quotation from a classic, like Hippocrates, may be more readily accessible if the reference is to book, chapter, and paragraph. In the reference to a periodical article the details are nearly the same: 1. Author's name with initials. 2. Title of article. 3. Title of periodical. 4. Place and date of publication. 5. Volume, or series and volume, 6. Page, or inclusive paging. When space is not at a premium the unabbreviated form of words of titles is a desirable precaution against error. Printing the title of the journal in italic or upper case type further serves to distinguish it from the title of the article. Some examples follow, each of which has a good list of references:

Harrower, H. R. The Typophysis or Pituitary Body. In his Practical Hormone Therapy. London: Bailliere, 1914. 287-319.

Janeway, T. C. Nephritic Hypertension. Harvey Lectures, 1912-1913. Phil., 1913. Ser. viii, 208-251.

Cotton, F. J., and Boothby, W. M. Intratracheal Insufflation Anaesthesia. Annals of Surgery. Phil., 1913. lvii, 43-63.

In such stenographic material, unlike reading matter, both grammar and context, clues to the correctness thereof, are absent, and the necessity for careful revision is apparent. However much verification be done in the course of composition, and that may well be considered, the bibliography should be gone over in toto in the proof. Really, the best way is to free one's self from the suggestion of copy, take the proof alone and consult the sources themselves, making the necessary corrections on the proof. Each detail of figure, letter, and mark of punctuation should be carefully scrutinized; spelling, date, volume number, page number, each should receive its own instant of attention. It is astonishing how many errors can be detected in a supposedly correct proof. Even then the influence of familiarity with the subject and with the articles consulted will slightly obscure the author's watchfulness. Some one unfamiliar with either will be more trustworthy in correcting the references.

"But why," some one asks, "is it not permissible, with all the catalogues and indexes we have, to say that Jones in 1900 did thus and so, or Jones, Journal of Medicine, 1900, said this and that?"

In the first instance, while you are saving space for yourself, you are probably wasting time for every reader of your article; in the second place, you are not saving enough to counterbalance its lack of efficiency; and in both cases the reference is of little worth in its collateral uses. In addition, you are mistaken in thinking that indexes are going to point out the article sought for at once, if at all. The indexes in magazines are themselves arguments for complete and correct reference. Indexes range all the way from the full and carefully made, like those in the Journal of the American Medical Association and the Journal of Experimental Medicine, through the slovenly and incomplete with elaborate but useless "table of contents," down to total absence of both "contents" and index. Atrophy and complete absence of index are too common in both journals and books; too common to speak well for medical journalism, and too common to trust medical literature to its mercy. The index may be lacking either from failure of the editors to have one made by a competent person, or from failure on their part to make the index an integral part of the magazine as published. Either cause is unexcusable and is decidedly bad for both journal and reader. These considerations lend emphasis to the demand for complete and correct references in bibliographies and to the slogan, Verify your references.

To the uninitiated all the details for a complete reference seem like redundance of information. To such the necessary points would be only the author, the journal, the year, the valume, and the page. In certain conditions, to save space, this brevity may be imposed upon one, but let it be only at command of the editor and when he brings forward proof of its necessity. For it must not be forgotten that the bibliography has uses beyond its connection with the article to which it is appended. A good bibliography is often used as a source of references without regard to the point of view of the paper itself. Frequently it is used by the student of the subject as a starting point for research rather than some of the more inclusive bibliographies like the Index-Catalogue. Such a list is also called upon to locate papers that other rfeerence books do not include. The more thoroughly the work on the bibliography is done, the greater is the service that it performs. As a finishing touch to the paper then, verify your references.

Though I have elsewhere (4) in greater detail taken up reasons for the inclusion of the several data in a reference, I wish to list some of them briefly here.

The author's name identifies the workman. The bibliography is an author index of the material referred to; it helps in this way in finding articles that indexes have omitted or that are too recent for any other index.

The title identifies the paper itself, especially when the author has written much on the subject. The title defines the scope of the paper without further search.

The place of publication (and in the case of a book, the name of the publisher) is of aid in identifying the publication.

The date of a book, journal, pamphlet, or other printed thing is, next to name of the author, the most important fact in scientific bibliography. It establishes the worth of the work as to its timeliness and its position in questions of priority. The date is a point of departure for finding other articles, later or earlier; it furnishes a clue to the scope of the paper.

The volume number reduces the quest to a single book; in most cases to a single sequence of pages. This is no small item in the case of a journal that publishes several volumes a year. The Biochemische Zeitschrift issues almost an even dozen volumes during the twelve months.

The page number directs the reader immediately to the point sought; while inclusive paging gives him in advance an idea as to the length of the article. When given, the page reference obviates the use of the index, which may be absent, or useless, or omit the entry one seeks.

For one reason or another the searcher is sometimes unable to find the original article that he needs; all that is available is an abstract. Let hi mgive a reference to the original as fully as possible from the information that he has, and then give also the citation of the abstract; something like this:

Murri, A., L'atassia nel cammino e nel nuoto. Riforma medica. Naples, Sept. 11, 1915, xxxi, No. 37. (Abstract in Journal A. M. A., Chic., 1915, lxv, 1496). From this it is seen that he has derived his reference from the Journal A. M. A. while the original article is to be found, so far as he can say, in Riforma medica. This method has its uses in quoting papers in other languages than English and in giving other sources in order that at least one may be available to the reader.

Give the title of the journal fro mwhich you quote. This seems as self-evident as an axiom, but it is not as foolish a request as it appears to be. This detail is omitted in references often enough to call for a few words. Instead of doing this simple thing, writers sometimes give only the name of the society before which the paper was read, an item of some meaning perhaps, but of little direct value in locating the paper. "Society of neurology," or Societe de neurologie, does not suggest to every one to look in the Revue neurologique rather than another neurological magazine.

This leads me to another point. Give authorities as they are printed, not as you would like to have them printed. If the title is in French, give it in French. A seeker who cannot read French will not spend time in digging up papers he cannot read. No one can object if, in addition to the title, a translation into English is given, especially if the other language is one that is generally unfamiliar, as Russian or Danish. In quoting other languages, however, great care must be exercised in transcribing names and titles of papers· To get the force of this take up a list of references in some foreign journal and observe those to English or American literature. Hilarity is likely to ensue. Parker Syms appears as Darker Syms; ''Diseases of the Pituitary Gland, by E. G. Fearnsides, M. A., M. D., B. C. Cantab., . .'' is given in a Jahrbuch as by ''E. C. Feamsider & B· C. Cantch.'' Verification is vexation, but it is the price of safety.

A common fault lies in taking a reference from another's bibliography as though it were thereby Gospel truth itself. Faith may remove mountains, but in science ye are known by your works. ''If the great Schmidt gives this reference, it is good enough for me.'' That is where trouble begins—or is continued; for the possibilities—nay, the probabilities—are that Professor Dr. Geh. Schmidt allowed an inexperienced assistant to round up the references; that another, equally untried, omitted to verify them in any way and in copying altered this one unconsciously; while a third let the printer still further maltreat it. The result, fair without but false within, may mean nothing even to Fetlock Jones or Doctor Swatson. Take no reference for granted. Verify the reference that your best friend gives you. Verify the reference that that your revered chief gives you. Verify, most of it, the reference that you yourself found and jotted down. To err is human, to verify is necessary.

Unless references are verified from the originals, marvelous are the results that are sometimes attained. Articles that mean nothing are ascribed to mythical authors; journals are quoted that never will be published, and dates are indicated that nóne of us will ever live to see. A classic example of bibliographic cocogenics is shown in the descent of the reference to a report of a case of urticaria by J. V. Hjelmman, published in a Finnish journal in 1899. This was abstracted in Progres medical, Paris, Jan. 27, 1900, 3 ser., xi, 60, but with no indication of its source in the Finnish journal. This French abstract was translated into English and printed in the Medical Bulletin, Phil., May, 1900, xxii, 175, but credited to "J. V. Hieleman, Progres medical" and without date, volume, or page. This translation was transferred bodily to the St. Louis Medical and Surgical Journal, August, 1900, lxxix, 96, credited to the same Hieleman, but now as originating in the "Bul. Med. and Surg." "J. C. J." transplanted this hardy annual to the October number, 1900, of the Journal of Cutaneous and Genito-Urinary Diseases, N. Y., 1900, xviii, 470, again deriving it from "Bull. Med. and Surg., 1900," but also giving due credit to the St. Louis Journal. Probably from the Journal of Cutaneous and G.-U. Diseases as his authority Hans Hubner added "Hielemann, Bull. Med. and Surg., 1900," to his bibliography which appeared with his article in the Archiv fur Dermatologie, Wien, 1906, lxxxi, on page 219. Seeing one of these latter references, who would turn for the original to Finska Lakaresallskapets Handlingar, Helsingform, 1899, xli, 1236-1241? And echo answers, "Who-who."

Verifying references means work, sometimes a good deal of work; but if your article and bibliography are to be worth anything they should be worth the work to make them so. Why not have the best? If they are not worth the work, they are not worth printing. While the directions herein enumerated are many and seem to make a counsel of perfection, the fact that these ideas have been acted upon by some writers shows that they are feasible. And yet these arguments for correct and complete references are not all that might be adduced. Every day, it seems to me, some newcomer turns up which reveals a new outlook on the uses of the bibliography. In every case the correct reference becames a time saver, a short cut through the hills of print. Wherefore I say again, Verify your references!

REFERENCES.

1. J. W. BURGON: Lives of Twelve Good Men, second edition, London, Macmillan, 1888, i, p. 73. 2. J. S. BILLINGS: Our Medical Literature, Transactions of the Seventh International Medical Congress, London, 1881, i, 54-70. Also: Boston Medical and Surgical Journal, 1881, cv, 217-222. See also his Medical Bibliography, Transactions of the Medical and Chirurgical Faculty of the State of Maryland, Baltimore, 1883, 58-80. 3. F. PLACE: Bibliographic Bones, Medical Pickwick, Saranac Lake, 1915, i, 82-84. 4. IDEM: Bibliographic Style in Medical Literature, Medical Record, N. Y., 1913, lxxxiii, 157-160.

New York Medical Journal.

---

### SOME PHASES OF AURAL DISEASE.

By S. MacCuen Smith, M. D.,

Professor of Diseases of the Ear in the Jefferson Medical College of Philadelphia.

Although many of us have agitated the question for a number of years, it is still apparent that an incredibly large percentage of the medical profession possesses a very meager appreciation of the danger-signals of aural afflictions.

Formerly, on account of lack of knowledge of the ear and its diseases, this apathetic attitude was comprehensible, but considering the widely disseminated educational campaign of recent years on pathologic etology, I feel that we have not achieved a success commensurate with our efforts.

Two principal reasons seem to be responsible for this indifferent attitude both on the part of some members of the profession and of the public. The latter is well illustrated by the old adage of our forefathers of some generations ago, born of their ignorance of the subject, that it is dangerous to interfere with a discharging ear; the second more important reason why this false impression still lives and struggles so tenaciously for its existence is due to the fact that some aurists yet adhere to the ultra-conservative method of treating acute suppurative diseases of he ear. We are all aware that occasionally a case, though presenting more or less alarming symptoms, will effect a spontaneous cure without operation. However safe this practice of "watchful waiting" may be in the hands of an expert, I believe it most dangerous to follow as a routine method of treatment even for the usual skilled aurist. The truth of the matter is that even with the aid of Roentgenography we are many times unable to estimate the exact degree of suppurative inflammation of the organ of hearing, or the direction in which this destructive carious erosion may proceed. Therefore, it seems most unwise to wait until grave symptoms, probably indicating an intracranial complication, present themselves before resorting to operative intervention.

I di not wish to be understood as advocating, for instance, a mastoid operation in cases of acute inflammation without making every effort in their non-surgical relief, but I do consider it unfortunate to wait purposely until there occurs a carious erosion through the cortex or through the tip, resulting in the characteristic swellings, before operation is advised.

Modern aseptic surgical otology has rendered the mastoid operation, if performed early, probably the most successful procedure in all surgery. Furthermore, early surgical intervention will not only prevent the various intracranial complications, in most instances, but will also almost assuredly preserve the hearing.

Some phases of this subject are forcibly illustrated by a few interesting cases which I will briefly recite.

In former years, when a diagnosis was doubtful, it was convenient for the medical profession to designate many such cases as malaria. In more recent times this malarial incubus has been banished, through the enlightenment of the popular mind, only to be replaced by the omnipresent influenza, a term at present quite acceptable to the public even though the condition be non-existent, and to-day we have the bacillus of

Friedlander arraigned at the bar as indiscriminately as was formerly the supposed miasma of malaria.

The following case of brain abscess well illustrates the error into which physicians are liable to fall, owing to incomplete diagnostic methods:

Male, sixty years of age, enjoyed exceptionally good health up to six weeks previous to operation, which occurred three years ago. Patient complained bitterly of general head pain, which was somewhat relieved by exerting severe pressure with his hands. This condition continued for a period of two weeks without additional manifestations. During this time he was advised to remain in bed, but refused compliance. At the end of two weeks he gradually became stuporous, with occasional lucid intervals, and so remained until the time of his death, four weeks later. Two weeks before he died he was removed to the hospital to be kept under observation. At the onset of his illness he was supposed to be suffering from influenza, and later, on account of having chills every second or third day, his malady was diagnosed as malaria, but a blood examination failed to confirm this suspicion.

I was walking past this patient's room with the resident physician, and my attention being attracted by his labored breathing, I remarked to the resident that it sounded like a patient suffering from a brain abscess. I was informed that a diagnosis had not been made, although the patient had been treated for the two diseases above mentioned. The resident asked me to examine the man, but as the case was a private one I was unable to comply with his request. Two days later, however, a spontaneous rupture of his right membrana tympani occurred, following by the same condition in the left ear early the next morning. At this time I examined the man, performed a simple mastoid operation on the right ear, and, on account of the great destruction, a radical operation on the left side. There were no exposures on the right side, but on the left we found a perisinus abscess formation, as well as a large carious exposure of the dura through the tegmen antri and tegmen tympani, well covered with granulation tissue, through the center of which pus escaped.

The interesting points in this case are that although the patient complained of severe head pains for a period of six weeks prior to the operation, he did not at any time locate this pain definitely in either ear. This no doubt was responsible for the failure to have an aural examination. The second point of interest is one of very common occurrence—the tendency, in the absence of an exact diagnosis, to pronounce an influenzal state the causative factor. It does not seem to be generally appreciated that confined pus within the tympanic or mastoid cavity will give symptoms simulating those of influenza, the result being that the ear, which is so frequently the only part of the organism involved, is entirely overlooked, showing the wisdom of a routine examination of the ear in a case of doubtful diagnosis. This is of special importance in children. Indeed, a failure to examine the ear would seem to be almost criminal neglect in many instances.

It is quite true that in this case the aural involvement may have been secondary to an influenzal infection, but, granting this, it would not explain the chills and semicomatose state which were continuous during the last week of the patient's life and latterly attributed to malaria.

Inasmuch as this patient, as above stated, did not complain of actual aural pain, it is not amiss to emphasize to the general profession that there are two distinct types of acute aural inflammation: one in which the symptoms are frank and very severe; the other type in which the symptoms referable to the ear are almost nil, even in the presence of most extensive destruction, and yet in this latter class the existence of aural disease can be determined on examination by an expert.

The second case well illustrates the folly of procrastination in some acute aural afflictions:

Male, seven years of age, contracted an acute suppurative otitis media, complicating a so-called "cold in the head." The ear was entirely overlooked for a period of two weeks, during which time the child suffered severaly from general head pains, no aural trouble being suspected until a discharge through a spontaneous rupture of the membrana tympani was discovered by the nurse, the temperature ranging meanwhile from 99° + to 104°. The spontaneous rupture was in the superior anterior quadrant, and was therefore enlarged and the middle ear aspirated. The pain and fever were lessened for two days only, and during the following week ranged as heretofore. It was impossible, on account of the child's fretfulness, to determine definitely the presence of tenderness on pressure over the mastoid process.

On several occasions I suggested to the attending physician the advisability of performing a mastoid operation. He, however, on account of the importance of the child, who was the sole heir to an enormous estate, hesitated to assent to this suggestion. On the ninth day following the spontaneous rupture there appeared a distinct drooping of the superior posterior wall. A mastoid operation was then urged and again refused, the same reason being given. The following day the child's mentality seemed to me to be somewhat cloudy. It was asserted, however, that this was due to a wakeful night. At this juncture I advised the medical attendant and the family that unless they consented to operative intervention, I would no longer assume the responsibility of the child's condition. This attitude resulted in another consultant being called, who in turn gave as his opinion that he saw no reason for immediate operation, although he felt that one migth be necessary eventually. This verdict was pleasing to both the child's physician and the family.

Eight days later I was summoned in great haste to see this child, being requested to bring my instruments with a view fo immediate operation. On arrival I found the patient had been semiconscious for two days, opisthotonos was present, several severe convulsions were reported, the temperature ranging as high as 106° +, and death ensued within the next half-hour, without the patient having the benefit of surgical intervention.

With this history it is needless to state that the boy succumbed to a diffuse, purulent meningitis, all of which, to my mind, would have been prevented had an early operation been permitted for the evacuation of the infectious material

The last case I shall outline shows another form of aural disease, not quite so common but just as grave:

Male, forty-one years of age, suffered from a chronic recurrent suppurative otitis media for thirty-eight years, the disease complicating an attack of measles at the age of three years. Until the last five years preceding operation the patient was not conscious of the slightest discomfort other than the annoyance of the discharge when present. During the last five years, however, he suffered almost constantly from what was termed a mild form of neuralgic headache, this at times becoming very severe. His general health was much impaired, to such an extent, indeed, that he was unable properly to attend to his large interests. This condition culminated in the patient being found unconscous in the drawing-room of a train between New York and Philadelphia. He was taken from the train to the hospital, where, on account of a foul-smelling discharge escaping from the ear and the absence of any other symptoms to account for his condition, I performed a radical mastoid operation. The cortex was ivory-like in density, the antrum decidedly small, and the sinus pushed far forward. The middle ear and antrum were well filled with considerable cholesteatomatous material and foul-smelling pus. The dura was exposed by carious erosion through the tegmen tympani, through an opening in which a quantity of pus escaped. While dilating forceds an additional amount of pus was evacuated from apparently the temporosphenoidal lobe.

This patiene completely recovered in due course and returned to his duties, and only recently expressed the opinion that he had never known what it was to feel really well and vigorous until after this operation. He has increased in weight and enjoys most excellent health.

Cases of neglected aural disease might be indefinitely enumerated not only in my practice, but in that of us all. My object, therefore, in presenting this elementary note is for the purpose of soliciting on the part of my colleagues a continuation of our educational programme.—The Therapeutic Gazette.

